# Effects of Dioxin Exposure on Brain Regional Volumes of Fathers from Birth Cohorts in Herbicide-Sprayed and Unsprayed Areas in Vietnam

**DOI:** 10.3390/toxics13090710

**Published:** 2025-08-23

**Authors:** Hai Minh Nguyen, Hoa Thi Vu, Thao Ngoc Pham, Tai Pham-The, Takashi Yokawa, Ryo Matsuda, Masafumi Nakamura, Muneko Nishijo, Yutaro Takahashi, Yoshikazu Nishino, Nghi Tran Ngoc, Hisao Nishijo

**Affiliations:** 1 Department of Diagnostic Imaging, Military Hospital 103, Vietnam Military Medical University, Hanoi 12108, Vietnam; nmhaidr@gmail.com; 2 Department of Military Hygiene, Vietnam Military Medical University, Hanoi 12108, Vietnam; 3 Department of Functional Diagnosis, Military Hospital 103, Vietnam Military Medical University, Hanoi 12108, Vietnam; phamngocthaovmmu@gmail.com; 4 Biomedical and Pharmaceutical Research Centre, Vietnamese Military Medical University, Hanoi 12108, Vietnam; taithuy@kanazawa-med.ac.jp; 5 Kobe Imaging Center, Bio View Co., Kobe 650-0047, Japan; yokawa@bioview.co.jp; 6 Hiyoshi Corporation, Omihachiman 523-8555, Japan; r.matsuda@hiyoshi-es.co.jp (R.M.); m.nakamura@hiyoshi-es.co.jp (M.N.); 7 Epidemiology and Public Health, Kanazawa Medical University, Uchinada 920-0293, Japan; ni-koei@kanazawa-med.ac.jp (M.N.); trem0ro25@gmail.com (Y.T.); ynishino@kanazawa-med.ac.jp (Y.N.); 8 Faculty of Clinical Medicine, Hanoi University of Public Health, Hanoi 12108, Vietnam; tnn@huph.edu.vn; 9 Department of Sport and Health Sciences, Faculty of Human Sciences, University of East Asia, Shimonoseki 751-8503, Japan

**Keywords:** dioxins, neuro imaging analysis, brain regional volume, residency, biological equivalency, Vietnamese men, Agent Orange

## Abstract

We previously reported that the fathers of the Bien Hoa birth cohort in Vietnam showed altered brain regional gray matter volumes, as measured by magnetic resonance imaging, and social anxiety traits associated with perinatal dioxin exposure. In the present study, we aimed to compare gray matter volumes and social anxiety scale scores between dioxin-exposed fathers in Bien Hoa and unexposed controls in an unsprayed area. Fat-based bioassay-toxic equivalency levels in serum were used to indicate dioxin exposure in adulthood. Results indicated that the longer Bien Hoa residency group (≥30 years) exposed to dioxins during the perinatal period and early childhood showed higher gray matter volumes in the right and left temporal lobes than controls. However, no significant differences in temporal lobe gray matter volumes were found between the shorter Bien Hoa residency group (<30 years) and controls. Furthermore, the longer, but not shorter, Bien Hoa residency group showed higher social–emotional subscale scores than controls. Additionally, fat-based bioassay-toxic equivalency levels were inversely correlated with gray matter volumes in several right temporal gyri. These findings suggest biphasic life stage-dependent adverse effects of dioxin exposure: perinatal dioxin exposure increases gray matter volumes, especially in the temporal lobe, which leads to neurodevelopmental disorders with socio-emotional disturbances, whereas dioxin exposure after brain development decreases cortical gray matter volumes, possibly leading to cognitive dysfunction.

## 1. Introduction

The Bien Hoa airbase, located in southern Vietnam, is the largest former United States airbase that was contaminated with dioxins originating from Agent Orange, containing 2,3,7,8-tetrachlorodibenzo-p-dioxin (TCDD), during the Vietnam War. Very high levels of TCDD were found in breast milk samples taken from nursing mothers in Bien Hoa City, ranging from 333 to 1832 pg/g lipid in 1970 and 133–266 pg/g lipid in 1973 [1,2]. Four decades after spraying ceased, dioxin levels in residents and areas surrounding the Bien Hoa airbase have been reported to remain elevated [3]. According to Van Luong et al. (2018), men who lived near the Phu Cat and Bien Hoa airbases had blood dioxin concentrations that were four to five times higher than those of an unsprayed area in northern Vietnam [4].

In areas around the Bien Hoa airbase, Nghi et al. (2015) reported high levels of 17 dioxin congeners, particularly TCDD, in maternal breast milk [5]. In 2015, we recruited infant–mother pairs who resided near the Bien Hoa airbase (i.e., the Bien Hoa birth cohort) and found an association between perinatal dioxin exposure and altered neonatal electroencephalography (EEG) power, coherence, neurodevelopment, and gaze behavior at 2 years of age [6,7]. Subsequently, fathers of the Bien Hoa birth cohort were invited to undergo assessments of blood dioxin levels and brain magnetic resonance imaging (MRI), which revealed alterations in regional brain volume (using voxel-based morphometry [VBM] analysis) that were associated with blood dioxin levels and estimated perinatal dioxin exposure [8]. In addition, affected brain regional volumes were associated with social anxiety disorder (SAD) symptoms, which indicated that these men in Bien Hoa exhibited both functional and morphological effects [9]. However, to date, brain regional volumes and SAD symptoms have not been compared between men exposed and unexposed to dioxins originating from Agent Orange.

Furthermore, we previously measured the biological equivalency (BEQ) levels of dioxin-like compounds in the serum of dioxin-exposed men in Bien Hoa who participated in the brain MRI examination [8,9] and unexposed men from a birth cohort in Hanoi, Vietnam, an unsprayed area, using the DR-EcoScreen bioassay and found a strong correlation between fat-based BEQ and World Health Organization’s toxic equivalents (TEQs). This demonstrated the utility of the DR-EcoScreen bioassay to discriminate exposed from unexposed individuals [10].

Therefore, in the current study, we aimed to investigate differences in brain regional volumes and SAD symptoms between exposed men in Bien Hoa and control subjects in an unsprayed area in Hanoi, Vietnam. Associations between BEQ levels measured with the DR-EcoScreen bioassay and brain regional volumes were also analyzed in all participants.

## 2. Materials and Methods

### 2.1. Study Subjects

In 2018, in the exposed group in communes near the Bien Hoa airbase, we measured blood dioxin exposure levels of 40 fathers of children from the Bien Hoa birth cohort whose newborn EEGs were analyzed in 2015 [7]. In 2019, these subjects were invited to undergo brain MRI examination; however, only 33 men (82.5%) underwent brain MRI examination (7 men were unavailable for assessment because of job obligations). Additionally, one person whose fat content was too low to use for correction of serum BEQ value was excluded. This resulted in 32 exposed men in the final data analysis. We also invited 32 fathers of a birth cohort in Hanoi, an unsprayed area in Vietnam, to undergo brain MRI examination and dioxin exposure assessment in 2023 as control subjects.

The characteristics of exposed men in Bien Hoa and unexposed men in the control group are shown in Table 1. Subjects in the exposed group were divided into two groups according to length of residency in Bien Hoa: <30 years (shorter Bien Hoa residency) and ≥30 years (longer Bien Hoa residency), since 30 years was the 75-percentile value of their length of residency. The shorter Bien Hoa residency group was significantly younger than the control group; however, there was no significant difference in age between the longer Bien Hoa residency group and the control group. Compared with the shorter Bien Hoa residency group (mean residence period: 13.9 ± 6.7 years, mean ± standard deviation (SD)), the longer residency group (mean residence period: 38.1 ± 4.1 years) were older and appeared to have spent most of their lives in Bien Hoa. No significant differences were found in educational level (years), height (cm), weight (kg), or body mass index between either the exposed group or controls. Approximately 90% of subjects were right-handed.

Written informed consent was obtained from all men according to a process reviewed and approved by the Health Departments of Bien Hoa City, Dong Nai prefectures and Hanoi city. The institutional ethics board for medical and health research involving human subjects at Kanazawa Medical University (No. I-424) and Military Hospital 103 of Vietnam Medical University (No. 107/CNChT-HDDD) approved the study design.

### 2.2. MRI Data Acquisition and Analysis

Brain MRI of the exposed men was performed using a Siemens Magnetom Trio Tim system 3-T scanner (Siemens, Erlangen, Germany) at the Department of Diagnostic Imaging in Dong Nai General Hospital, Vietnam. We collected high-resolution T1-weighted images with good gray and white matter contrast using the following imaging parameters: repetition time = 1520 ms, echo time = 2.07 ms, flip angle = 9°, slice thickness = 0.9 mm, 192 slices, and field of view = 230 × 230 mm. The control group underwent brain MRI scans on a Siemens Magnetom Essenza 1.5-T scanner (Siemens, Erlangen, Germany) at the Department of Diagnostic Imaging in 103 Military Hospital, Vietnam using similar imaging parameters to those used for the exposed group.

The MRI image analysis method has been described previously [8]. In brief, we used the Computational Anatomy Toolbox (CAT12, vCAT12.7-RC1; Structural Brain Mapping Group, Jena University Hospital, Jena, Germany; Available online: http://dbm.neuro.uni-jena.de/cat/) (accessed on 14 December 2021) in the Statistical Parametric Mapping 12 software package (SPM12; The Wellcome Centre for Human Neuroimaging, London, UK; Available online: https://www.fil.ion.ucl.ac.uk/spm/software/spm12/) (accessed on 14 December 2021) in a MATLAB environment (R2020a:The Mathworks, Inc., Natick, MA, USA) to preprocess the images using the standard protocol (Available online: http://neuro-jena.github.io/cat12-help/) (accessed on 14 December 2021). The gray matter, white matter, and cerebrospinal fluid components of all T1-weighted MRI images were segmented following affine registration and rough bias correction [11]. DARTEL was used to normalize the segmented pictures to standard Montreal Neurological Institute 152 space, and an isotropic Gaussian kernel (full width at half maximum = 8 mm) was used for smoothing. Using the estimation mean value within the region of interest (ROI) function in CAT12, we obtained the gray matter volume of ROIs based on the Neuromorphometrics atlas. The total volume of the brain lobules and gyri that make up the lobes was used to determine the volume of the frontal and temporal lobes [12,13].

### 2.3. Dioxin Exposure Assessment Using the DR-EcoScreen Bioassay

Levels of dioxin-related compounds were estimated in subjects’ serum samples using the DR-EcoScreen bioassay, which is an improved version of the Dioxin-Responsive Chemically Activated LUciferase Expression bioassay. The DR-EcoScreen bioassay is based on genetically engineered DR-EcoScreen cells, which are mouse hepatoma Hepa1c1c7 cells stably transfected with a reporter plasmid containing seven copies of a dioxin-responsive element [14]. The analysis was conducted at Hiyoshi Corporation, Shiga, Japan. The protocols used for the DR-EcoScreen bioassay and analytical validity have been described elsewhere [10]. The DR-EcoScreen bioassay yields bioassay TEQs that indicate the total toxic potency of all dioxins and dioxin-like congeners that exhibit affinity to the aryl hydrocarbon receptor (AhR) in each sample. Levels of dioxin-related compounds are expressed as pg BEQ per g of fat (i.e., fat-based BEQ values).

The mean BEQ values in the shorter and longer Bien Hoa residency groups and the control group are shown in Table 1. The shorter Bien Hoa residency group showed higher fat-based BEQ than the longer Bien Hoa residency group, although the difference in BEQ levels was not significant between the two groups. This unexpected result was consistent with our previous study reporting that blood dioxin levels measured by instrumental analysis, in Bien Hoa fathers, were not correlated with residence period, but associated with their occupation, particularly jobs related with Bien Hoa airbase such as solders [8]. These findings suggest that fat-based BEQ levels in this study reflected recent exposure to dioxins during occupational work in adulthood, but not cumulative long-term exposure to sprayed herbicides in the soil due to the Vietnam War.

### 2.4. Social Anxiety Symptoms Assessment

SAD is a type of anxiety disorder that manifests as extreme fear or anxiety around strangers or in social settings where they might be watched closely by others [15]. We used the social anxiety questionnaire for adults (SAQ-A30), which is a multidimensional self-reported tool that has been used globally to measure social anxiety with high intercultural validity [16,17]. The 30 items on the SAQ-A30 are divided into five factors: F1: speaking in front of others or having conversations with persons in positions of power; F2: interacting with people of the opposite sex; F3: expressing dissatisfaction, contempt, or disapproval in an aggressive manner; F4: criticism and embarrassment; and F5: interacting with strangers. Each item is rated on a 5-point Likert scale for level of unease, stress, or nervousness ranging from 1 (not at all or very slight) to 5 (very high or extremely high) [16].

### 2.5. Statistical Analysis

SPSS version 22.0 (IBM; Armonk, NY, USA) was used for all statistical analyses. We analyzed the difference in regional gray matter volume among the three regional groups (i.e., shorter and longer Bien Hoa residency groups and the control group) using a general linear model after adjusting for confounding factors, such as age, height, and fat-based BEQ value. Because altered gray matter volumes in the frontal and temporal lobes have previously been shown to be associated with perinatal dioxin exposure [9], we examined only the frontal and temporal lobes. In addition, if the partial correlation coefficient (B) between regional gray matter volume and the fat-based BEQ value was significant in each analysis, we performed correlation analysis using Spearman’s ρ and the standardized β of the fat-based BEQ value, adjusted for age and height, for the Bien Hoa residency group and the control group.

We also compared the adjusted mean SAQ-A30 scores between the shorter and longer Bien Hoa residency groups and the control group using a general linear model, with the covariates of age, fat-based BEQ values, and educational level. For all tests, *p* < 0.05 was considered significant.

## 3. Results

### 3.1. Effects of Dioxin Exposure on Frontal and Temporal Gray Matter Volumes

We first compared the mean gray matter volumes of the frontal and temporal lobes among the three regional groups in the analysis of covariance (ANCOVA) model with covariates including fat-based BEQ values. The results of Bien Hoa residency groups compared with control group are shown in Table 2. The mean gray matter volumes of both the right and left temporal lobes were significantly higher in the longer Bien Hoa residency group than in the control group. However, there was no significant difference in the temporal lobe volume between the shorter Bien Hoa residency group and the control group. In the frontal lobe, there was no significant difference in gray matter volume between the shorter or longer Bien Hoa residency group and the control group.

A correlation analysis between fat-based BEQ values and temporal lobe volume for the Bien Hoa residency and control groups was not performed because the partial correlation coefficient B of the fat-based BEQ value in ANCOVA analysis was not significant in either the left or right temporal lobes.

### 3.2. Effects of Dioxin Exposure on Gray Matter Volumes in the Gyri of the Temporal Lobes

#### 3.2.1. Associations Between Residency in Bien Hoa and Gyrus Volumes of the Temporal Lobes

To investigate which gyri contributed to the higher temporal lobe volume, we compared the gray matter volume of the gyri of the temporal lobes between each Bien Hoa residency group and control group in group comparisons of the ANCOVA analysis (Table 3). The gray matter volume of each gyrus of the longer Bien Hoa residency groups was higher than that of the control group: increases in gray matter volumes in the left inferior temporal gyrus, right middle temporal gyrus, and left and right temporal pole in the longer Bien Hoa residency group were statistically significant under Bonferroni correction. The shorter Bien Hoa residency group, however, showed no significant difference in gray matter volumes in any gyri in both hemispheres compared with the control group.

#### 3.2.2. Associations Between Fat-Based BEQ Levels and Gyrus Volumes of the Temporal Lobes

In the ANCOVA, the partial correlation coefficients (B), representing decreases in regional brain volume for 1 (pg/g fat) of fat-based BEQ, showed negative values in the inferior temporal gyrus, middle temporal gyrus, parahippocampal gyrus, and temporal pole in the right hemisphere of the longer Bien Hoa residency group in Table 3). This suggested an inverse effect of BEQ levels on regional brain volumes.

**Figure 1:**
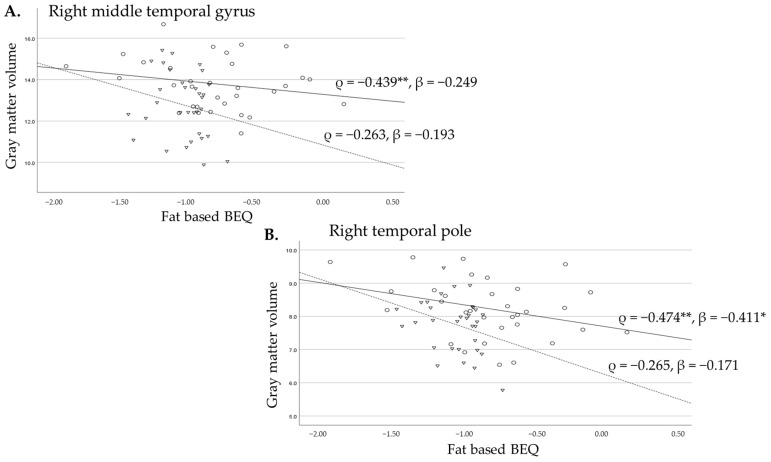
These are scatter plots to show the difference in the relationship between the gyrus volumes and fat-based BEQ levels for the right middle temporal gyrus (**A**) and the right temporal pole (**B**) between the men exposed to dioxins indicated by circles and a straight line and unexposed men indicated by triangles and a broken line. ρ: Spearman’s correlation coefficients; β: standardized beta adjusted for age and height, *: *p* < 0.05, **: *p* < 0.01.

**Figure 2:**
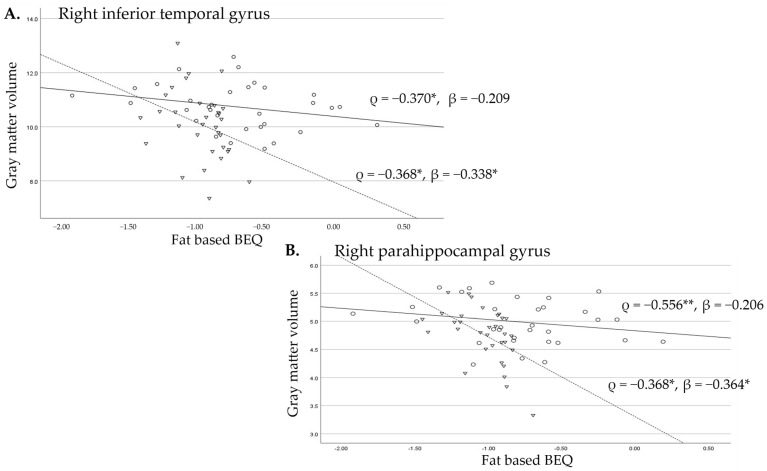
These scatter plots show the difference in the relationship between the gyrus volumes and fat-based BEQ levels for the right inferior temporal gyrus (**A**) and the right parahippocampal gyrus (**B**) between the men exposed to dioxins indicated by circles and a straight line and unexposed men indicated by triangles and a broken line. ρ: Spearman’s correlation coefficients; β: standardized beta adjusted for age and height, *: *p* < 0.05, **: *p* < 0.01.

The scatter plots for the regional volume of each gyrus and BEQ levels, with sample markers according to residency (circles: exposed Bien Hoa area; triangles: unexposed control area), correlation coefficients of Spearman’s ρ and standardized β (adjusted for age and height) for each area, and regression lines (straight lines: exposed Bien Hoa area; broken lines: unexposed control area) are provided in Figure 1 and Figure 2. These figures illustrate the differences in the relationship between the right temporal lobe gyrus volume and BEQ levels between the men exposed and unexposed to dioxins. Although the middle temporal gyrus and temporal pole brain volumes were significantly inversely correlated with fat-based BEQ levels in Bien Hoa residents (Figure 1A,B), the association between them, indicated by the β value representing the change in the standardized regional brain volume for one standard deviation change in the standardized BEQ levels, was significant only for the temporal pole after adjusting for confounding factors (Figure 1B). In controls, these brain regions showed no significant associations (Figure 1A,B).

In contrast, for the right inferior temporal and parahippocampal gyri, we found no significant associations in the Bien Hoa residents, after adjusting for confounding factors, whereas brain volumes were significantly lower with higher fat-based BEQs in the controls, even after adjusting for confounders (Figure 2A,B).

Because the partial inverse correlation with fat-based BEQ value was significant in the ANCOVA, we then analyzed the standardized β values for each area and obtained results similar to those obtained for the right temporal pole. Specifically, we found that a lower gray matter volume was significantly associated with higher fat-based BEQ levels (β = −0.391, *p* < 0.05) in Bien Hoa residents but not controls (β = −0.142, *p* = 0.453). These results suggest that higher fat-based BEQs associated with dioxin exposure originating from Agent Orange were related to a lower bilateral temporal pole gray matter volume.

### 3.3. Effects of Dioxin Exposure on SAD Symptoms

We compared the mean scores of the five factors (F1 to F5) and the total SAQ-A30 scores among the three residency groups, after adjusting for age, fat-based BEQ values, and educational level (Table 4). The adjusted mean score for F4 (criticism and embarrassment) was significantly higher in the longer Bien Hoa residency group than in the controls in the model with covariates of age, fat-based BEQ values, and educational level (*p* < 0.05), as well as in the models with no covariates (*p* < 0.01) and with covariates of age and fat-based BEQ values (*p* < 0.01). However, there were no significant differences in the other factors or total scores between the longer or shorter Bien Hoa residency groups and the control group. In addition, the ANOVA revealed no significant associations between fat-based BEQ values and any of the SAQ-A30 scores in any of the groups.

## 4. Discussion

### 4.1. Effects of Dioxin Exposure on Brain Gray Matter Volumes (Morphological Effects)

In the present study, higher gray matter volumes of the bilateral temporal lobes were observed in the longer Bien Hoa residency group than in the unexposed men. However, no significant difference was found in the gray matter volume of the temporal lobe between the shorter Bien Hoa residency group and the unexposed men. These results indicate that dioxin exposure originating from Agent Orange predominantly alters temporal lobe volumes, which is consistent with our previous study targeting Bien Hoa residents [9]. In particular, the middle temporal gyrus and temporal pole in the right temporal lobe of the longer Bien Hoa residency group, showed significantly higher gray matter volume than controls, indicating a meaningful impact of dioxin exposure due to the high enough effect size (partial eta squared = 0.203 and 0.223, respectively). We also detected significantly higher volumes of the inferior temporal gyrus and temporal pole in the left temporal lobe in the longer Bien Hoa residency group, although the effect size was a little smaller than those of gyri in the right lobe (partial eta squared = 0.166 and 0.199, respectively).

These results are consistent with our previous reports that showed that increased brain volumes of the superior temporal gyrus and temporal pole contribute to enlargement of the temporal lobe in men with perinatal dioxin exposure [9]; moreover, a considerable proportion of individuals in the longer Bien Hoa residency group had been exposed to dioxins perinatally.

We also noted that gray matter volumes in the gyri of the temporal lobe, particularly the temporal pole, were significantly lower in those with higher fat-based BEQ levels, which correlated with TEQ-polychlorinated dibenzo-p-dioxins (PCDDs)/polychlorinated dibenzo-p-furans (Fs)/dioxin-like polychlorinated biphenyls (dl-PCBs) in blood owing to dioxin exposure during adulthood [8]. Together, these results suggest a biphasic effect of dioxin exposure on brain regional volume: an enlargement due to dioxin exposure during the developmental period in early life and a reduction due to dioxin exposure during adulthood.

### 4.2. Effects of Dioxin Exposure on SAD Symptoms (Functional Effects)

The comparison of social anxiety scores among the three residency groups revealed significantly higher scores for the F4 subscale, one of the social–emotional subscales, in the longer Bien Hoa residency group than in the controls (partial eta squared = 0.146 indicating not small effect size); moreover, they exhibited higher gyrus volumes in several gyri of the temporal lobe. Furthermore, we observed higher F4 scores in men with perinatal dioxin exposure, 75% of whom had resided in Bien Hoa for more than 30 years, than in men without perinatal dioxin exposure. This is in line with the positive correlation between F4 scores and right middle temporal gyrus volume reported previously [9] in the same Bien Hoa men. In our previous study, we also reported higher scores on the F2 subscale, another social–emotional subscale, associated with the right fusiform gyrus, parahippocampal gyrus, and temporal pole gray matter volumes, in Bien Hoa residents than in controls [9]. Taken together, these findings suggest that dioxin exposure impacted brain development in early life, which led to an increase in social-emotional symptoms in adulthood.

To confirm that the associations between brain regional volumes and social anxiety symptoms were found in only the dioxin-exposed subjects, we analyzed the adjusted associations between all SAQ-A30 scores and gyri volumes in the right temporal lobe in the controls. We found no significant associations except an inverse correlation between F5 scores (interactions with strangers) and middle temporal gyrus volume (data not shown). These findings suggest that significant associations between gyri volumes in the right temporal lobe and social–emotional symptoms (F2 and F4) are ascribed to dioxin exposure in early life in men leading to brain morphological changes with temporal lobe enlargement (see the section below for more information).

### 4.3. Neurotoxic Effects of Dioxins on the Developing Brain

In Bien Hoa, we found that lower EEG power in neonatal infants measured 2 days after birth was associated with dioxin levels in maternal breast milk, which led to lower language developmental scores at 2 years of age [6]. This suggested that dioxin effects on the brain during the fetal period impact brain development after birth. Pham et al. (2021) followed up the same infants with altered neonatal EEG findings and observed atypical gaze behavior at 2 years of age and increased autistic symptoms at 3 years of age [7].

Previous morphological studies in individuals with autism have identified brain regions specific to social communication and autistic symptoms. Zahn et al. [18] reported that the anterior region of the superior temporal gyrus plays a key role in social cognition by providing abstract conceptual knowledge of social behaviors. According to Grecucci et al. (2016) [19], there is an autism-specific structural network that includes several gyri of the temporal lobe, such as the superior and middle temporal gyri. These regions are involved in social cognition and high-level visual processing, and in individuals with autism, these areas show gray matter abnormalities. Our results and previous findings suggest that dioxin exposure impacts the volumes of temporal gyri, which results in increased autism spectrum disorder symptoms. Furthermore, the neurodevelopmental traits of autism are one of the risk factors for SAD symptoms [20]. Therefore, these findings suggest that dioxin exposure impacts fetal brain development (e.g., enlargement of the gray matter volumes of temporal gyri), which subsequently leads to the development of neurodevelopmental disorders such as autism and social–emotional deficits during childhood, which may be detected later in adulthood via MRI and specific psychological examinations to assess social communication skills.

### 4.4. Neurotoxic Effects of Dioxins on the Adult Brain

In the current study, lower gyri volume was associated with higher dioxin exposure during adulthood, as indicated by fat-based BEQ values, which are highly correlated with blood levels of TEQ-PCDD/Fs and TEQ-PCDD/Fs/dl-PCBs. Consistent with the present results, Korean Vietnam War veterans presumed to have been exposed to Agent Orange (including high levels of TCDD) in adulthood have been shown to exhibit progressive brain atrophy on repeated brain MRI examinations, particularly in the ventrolateral prefrontal lobe and the entire temporal lobe [21], leading to an increased risk of dementia [22]. Furthermore, Martinez et al. [23] analyzed the adjusted risk of a dementia diagnosis of US veterans exposed to Agent Orange during the Vietnam War, and the risk ratio for veterans with Agent Orange exposure was approximately double that of veterans without exposure.

Given that BEQ levels indicate the total toxic potency of all dioxins and dioxin-like congeners mediated by the AhR, increased AhR activity induced by dioxin exposure may contribute to the smaller regional brain volumes observed in the middle-aged adults in the current study as well as in older adults with Alzheimer’s disease [24]. Previous animal and in vitro studies have demonstrated that the AhR signaling pathway is involved in various aging hallmarks in the brain, such as glial cell activation and inflammation, and is thought to progress brain aging [25]. However, in human studies, very few studies have investigated brain morphology, cognitive function, and AhR activity, particularly in dioxin-exposed subjects. Therefore, we plan to investigate the effects of dioxin-indicated fat-based BEQs on brain volume and cognitive function in older residents including women who were not investigated in the current brain MRI study.

### 4.5. Limitations

There are several limitations of the current study. First, the sample size was small as an epidemiological study. In particular, residents extremely heavily exposed to Agent Orange during or just after the Vietnam War were not included. Second, we did not analyze female subjects (see above). Further studies are required to increase the number of subjects, including older men and women. Accordingly, we are planning such additional studies.

## 5. Conclusions

Compared with unexposed subjects, those residing in Bien Hoa who had been exposed to dioxins originating from Agent Orange during childhood had significantly higher temporal lobe gray matter volumes and SAD symptom scores (criticism and embarrassment). Furthermore, fat-based BEQ levels in adulthood were inversely correlated with gray matter volumes in several right temporal lobe gyri, which indicated a link between lower brain volume and higher dioxin-like activity.

Consistent with the above findings, it has been suggested that dioxins exert a biphasic effect: dioxins, depending on their concentrations, may lead to neuronal dendritic overgrowth and damage [26,27], which may in turn lead to increases and decreases in brain volumes. Furthermore, it has been proposed that AhR displays antagonistic pleiotropy, in which AhR is involved in both development in early life stages and detrimental pathology in later life stages [28].

These findings suggest that the effects of dioxin exposure on neurite growth and maintenance are biphasic and dependent on life stages; dioxin exposure in early life may result in neurite overgrowth in the temporal lobe, leading to the development of neurodevelopmental diseases, whereas dioxin exposure after brain development may lead to suppression of neurite outgrowth and maintenance, resulting in the onset of age-related neurodegenerative diseases.

## Figures and Tables

**Table 1:** Characteristics of the subjects and BEQ levels between exposed men with different residency in Bien Hoa and unexposed controls in Hanoi.

Residency Groups	Controls (*n* = 32)		Shorter BH Residency (*n* = 22)			Longer BH Residency (*n* = 10)		
	Mean	SD	Mean	SD	*p*-Value	Mean	SD	*p*-Value
Age (years)	39.3	4.2	34.1	5.7	0.001	38.3	5.4	0.795
Education (years)	13.4	3.2	12.2	3.3	0.298	11.5	3	0.184
Height (cm)	165.6	5.2	164.9	4.7	0.818	166.5	5.7	0.867
Weight (Kg)	64.4	8.6	65.2	9.4	0.930	68.9	8.7	0.295
BMI	23.4	2.4	24	3.1	0.713	24.8	2.7	0.274
Fat-based BEQ (pg/g fat)	53.1	1.8	148.8	2.5	<0.001	106.5	2.1	0.020

BEQ: biological equivalency, BH: Bien Hoa, *n*: number of the subjects, SD: standard deviation.

**Table 2:** Comparisons of the adjusted brain volumes of frontal and temporal lobes among the three regional groups.

Residency Groups	N	Mean	SD	Adj Mean	95%CI		*p*
Left frontal lobe							
Control	32	93.9	8.8	94.3	91.3	97.3	ref
Shorter BH residency	22	94.7	7.9	93.9	90.2	97.7	ns
Longer BH residency	10	99.3	5.1	99.6	94.9	104.2	ns
Right frontal lobe							
Control	32	94.5	8.7	94.8	91.8	97.7	ref
Shorter BH residency	22	95.7	7.7	95.3	91.6	99.0	ns
Longer BH residency	10	100.7	5.2	100.8	96.3	105.4	ns
Left temporal lobe							
Control	32	57.2	5.5	56.9	55.1	58.6	ref
Shorter BH residency	22	58.3	4.6	58.6	56.4	60.8	ns
Longer BH residency	10	61.6	2.8	61.8	59.1	64.5	*
Right temporal lobe							
Control	32	56.8	5.3	56.4	54.8	58.0	ref
Shorter BH residency	22	58.6	4.2	59.1	57.1	61.1	ns
Longer BH residency	10	62.5	3.2	62.5	60.1	65.0	**

N: number of subjects; SD: standard deviation; Adj Mean: mean adjusted with age, height, and fat-based BEQ value; 95%CI: 95% confidence interval; BH: Bien Hoa; *p*: *p*-value; ref: reference; ns: not significant; *: *p* < 0.05; **: *p* < 0.01.

**Table 3:** Comparisons of the adjusted brain gyri volumes of temporal lobes among the three regional groups.

	Left Hemisphere					Right Hemisphere				
Residency Groups	N	Adj Mean	95%CI		*p*	N	Adj Mean	95%CI		*p*
Superior Temporal Gyrus										
Control	32	11.0	10.6	11.4	ref	32	10.9	10.5	11.3	ref
Shorter BH residency	22	11.3	10.8	11.8	ns	22	10.8	10.3	11.3	ns
Longer BH residency	10	11.9	11.3	12.5	ns	10	11.9	11.3	12.5	ns
Fusiform Gyrus										
Control	32	7.3	7.1	7.6	ref	32	7.3	7.1	7.6	ref
Shorter BH residency	22	7.5	7.1	7.8	ns	22	7.5	7.2	7.8	ns
Longer BH residency	10	7.7	7.3	8.2	ns	10	8.0	7.6	8.3	ns
Inferior Temporal Gyrus										
Control	32	9.6	9.3	10.0	ref	32	9.9	9.5	10.3	ref
Shorter BH residency	22	10.2	9.7	10.6	ns	22	10.8	10.3	11.4	ns
Longer BH residency	10	10.8	10.2	11.4	*	10	11.1	10.5	11.8	ns
Middle Temporal Gyrus										
Control	32	13.2	12.6	13.7	ref	32	12.6	12.1	13.1	ref
Shorter BH residency	22	14.0	13.3	14.7	ns	22	13.8	13.2	14.5	ns
Longer BH residency	10	14.0	13.1	14.8	ns	10	14.4	13.6	15.2	*
Parahippocampal Gyrus										
Control	32	4.8	4.6	4.9	ref	32	4.7	4.5	4.9	ref
Shorter BH residency	22	4.9	4.7	5.1	ns	22	5.0	4.8	5.2	ns
Longer BH residency	10	5.1	4.9	5.4	ns	10	5.1	4.9	5.4	ns
Temporal Pole										
Control	32	7.8	7.4	8.2	ref	32	7.7	7.4	8.0	ref
Shorter BH residency	22	7.8	7.3	8.3	ns	22	8.1	7.7	8.4	ns
Longer BH residency	10	9.1	8.4	9.7	*	10	8.8	8.3	9.2	**

N: number of the subjects; SD: standard deviation; Adj Mean: mean adjusted with age; height; and fat-based BEQ value; 95%CI: 95% confidence interval; BH: Bien Hoa; *p*: *p*-value; ref: reference; ns: not significant; *: *p* < 0.05; **: *p* < 0.01 (Bonferroni-corrected for multiple comparisons).

**Table 4:** Adjusted comparisons of the social anxiety questionnaire (SAQ-A30) scores among the three regional groups.

Social Anxiety Questionare (SAQ-A30)	Controls				Shorter BH Residency				Longer BH Residency				*p*
	Mean	Adj Mean	95%CI		Mean	Adj Mean	95%CI		Mean	Adj Mean	95%CI		
Speaking in public (F1)	14.5	14.5	12.4	16.6	14.6	14.5	11.9	17.1	14.0	14.1	10.9	17.3	ns
Interaction with the opposite sex (F2)	10.5	10.2	8.6	11.7	12.1	12.5	10.6	14.4	12.7	12.8	10.5	15.1	ns
Assertive expression of annoyance, disgust or displeasure (F3)	12.7	12.2	11.0	13.5	12.9	13.4	11.9	14.9	12.2	12.1	10.3	14.0	ns
Criticism and embarrassment (F4)	11.3	11.2	10.1	12.3	12.1	12.2	10.8	13.6	14.4	14.4	12.7	16.1	*
Interaction with strangers (F5)	14.0	14.0	12.8	15.3	15.1	15.2	13.6	16.8	14.6	14.4	12.4	16.3	ns
Total	63.0	62.0	56.8	67.2	67.0	68.2	61.8	74.6	68.5	68.5	60.6	76.4	ns

N: number of subjects; adj Mean: adjusted mean; CI: confidence interval. BH: Bien Hoa; *: *p* < 0.05 compared with controls after adjusting with age; education years; and fat-based BEQ levels.

## Data Availability

The raw data supporting the conclusions of this article will be made available by the authors on request.
